# Sentiment analysis of user feedback on the HSE’s Covid-19 contact tracing app

**DOI:** 10.1007/s11845-021-02529-y

**Published:** 2021-02-18

**Authors:** Kaavya Rekanar, Ian R. O’Keeffe, Sarah Buckley, Manzar Abbas, Sarah Beecham, Muslim Chochlov, Brian Fitzgerald, Liam Glynn, Kevin Johnson, John Laffey, Bairbre McNicholas, Bashar Nuseibeh, James O’Connell, Derek O’Keeffe, Mike O’Callaghan, Abdul Razzaq, Ita Richardson, Andrew Simpkin, Cristiano Storni, Damyanka Tsvyatkova, Jane Walsh, Thomas Welsh, Jim Buckley

**Affiliations:** 1 Lero - The Irish Software Research Centre, Tierney Building, University of Limerick, Limerick, Ireland; 2 Department of Computer Science and Information Systems, University of Limerick, Limerick, Ireland; 3 Applied Psychology, University College Cork, Cork, Ireland; 4 School of Medicine, University of Limerick, Limerick, Ireland; 5 Health Research Institute, University of Limerick, Limerick, Ireland; 6 College of Education & Health Sciences, University of Limerick, Limerick, Ireland; 7 School of Medicine, National University of Ireland Galway (NUIG), Galway, Ireland; 8 School of Mathematics, Statistics and Applied Mathematics, National University of Ireland Galway, Galway, Ireland; 9 School of Psychology, National University of Ireland Galway, Galway, Ireland

**Keywords:** Contact tracing, Coronavirus, COVID-19, mHealth, Public opinion, Sentiment analysis

## Abstract

**Background:**

Digital Contact Tracing is seen as a key tool in reducing the propagation of Covid-19. But it requires high uptake and continued participation across the population to be effective. To achieve sufficient uptake/participation, health authorities should address, and thus be aware of, user concerns.

**Aim:**

This work manually analyzes user reviews of the Irish Heath Service Executive’s (HSE) Contact Tracker app, to identify user concerns and to lay the foundations for subsequent, large-scale, automated analyses of reviews. While this might seem tightly scoped to the Irish context, the HSE app provides the basis for apps in many jurisdictions in the USA and Europe.

**Methods:**

Manual analysis of (1287) user reviews from the Google/Apple playstores was performed, to identify the aspects of the app that users focused on, and the positive/negative sentiment expressed.

**Results:**

The findings suggest a largely positive sentiment towards the app, and that users thought it handled data protection and transparency aspects well. But feedback suggests that users would appreciate more targeted feedback on the incidence of the virus, and facilities for more proactive engagement, like notifications that prompt users to submit their health status daily. Finally, the analysis suggests that the “android battery” issue and the backward-compatibility issue with iPhones seriously impacted retention/uptake of the app respectively.

**Conclusion:**

The HSE have responded to the public’s desire for targeted feedback in newer versions, but should consider increasing the app’s proactive engagement. The results suggest they should also raise the backward compatibility issue, regarding older iPhones, with Apple.

## Introduction

As the coronavirus (Covid-19) continues to spread globally, governments and public health institutions look to contact tracing to help isolate and contain outbreaks. The more traditional manual contact tracing approach initially adopted in Ireland is both time and resource intensive, and may struggle to identify all contacts quickly enough, before they cause further transmission. In contrast, the efficiency and responsiveness of a digital approach using the proximity sensors in smartphone devices has the potential to limit delay and catch a greater number of contacts [1].

Key to the effectiveness of these digital solutions is the take-up of the apps across the population: A study from the UK [2] recommends that the epidemic could be suppressed by 56% of the population using a contact tracing app. Given that, in Ireland, 3.62 million people use a smartphone [3] from a population of 4.9 million (73%), the UK recommendation suggests that an app user base of 2.78 million (76.7% of smartphone users) would suppress the virus in Ireland. Currently, the app has 1.3 million active users (according to the information page on the app itself).

### Motivation

Analysis of the public’s response to the initial release of the HSE Contact Tracker app [4] can help guide the system’s evolution towards greater uptake and ongoing use of the app, in order to fight transmission of Covid-19 in Ireland. Specifically, such an analysis can inform on how users would like the app to evolve over its lifetime. The voluntary nature of app usage, combined with the requirement for a critical mass of users across the country to make the app effective, makes such feedback a crucial tool in the campaign to defeat the spread of the virus.

To that end, this research [5, 6] manually analyzes all app reviews of the HSE app on the AppStore [7] and Google Play [8], using seven different aspects of interest: General Characteristics, Usability, Functional Effectiveness, Performance, Data Protection, Autonomy (of users), and Overall (generic comments). This analysis focuses on “positive” and “negative” sentiments (opinions) expressed by the user under each of these aspects, in order to identify areas well received, and to target areas where future releases of the app could be refined. Additionally, the manual analysis provides us with insights that can be leveraged for subsequent, large-scale, automated analysis of Contact Tracing apps’ user reviews.

### HSE app review

**Fig. 1:**
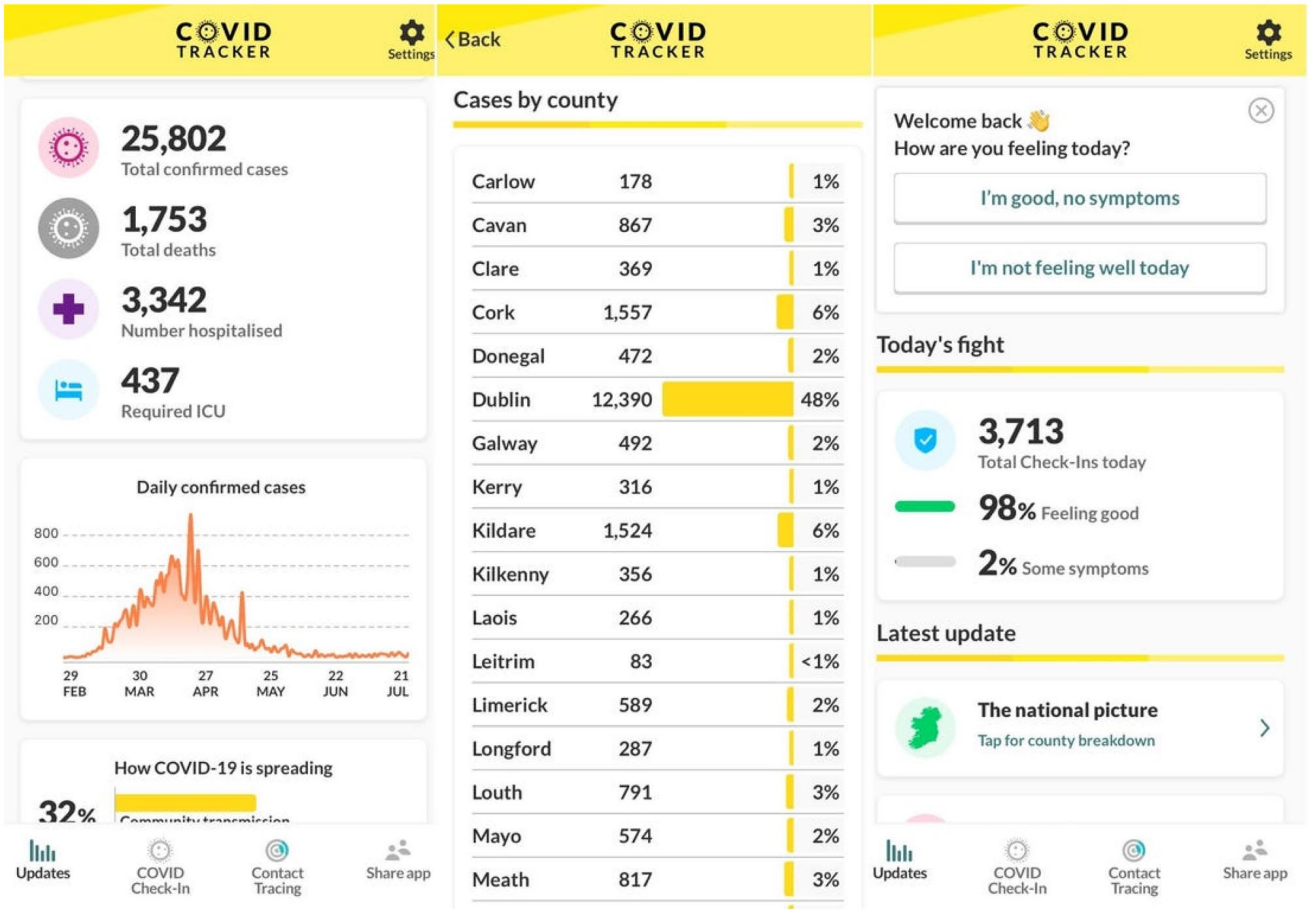
Screen captures of the HSE Covid Tracker app

Ireland’s Health Service Executive released the COVID Tracker app (see Fig. 1), developed by NearForm, across the Apple and Google online app stores in early July 2020.

Built on the Google and Apple Exposure Notification API (GAEN), it uses Bluetooth and anonymous IDs to log any other phone (with the app) it is in close contact with—tracking the distance and the time elapsed. Every 2 h, the app downloads a list of anonymous IDs that have been shared with the HSE by other users that have tested positive for Covid-19. If a user has been closer than 2 m for more than 15 min with any of these phones, they will get an alert that they are a close contact. The app runs in the background.

Beyond the core contact tracing functionality lies additional voluntary self-reporting functionality: Users can choose to log daily health status or symptoms via the Check-In option, and also to share their age, group, sex, and locality. Also optional is the ability to share a contact phone number so the HSE can contact them.

### Research questions

In order to inform the ongoing development of the HSE Covid Tracker app, the following two research questions were formulated:

1. How do users perceive the HSE Covid Tracker app version 1.0.0?
2. What are the prevalent issues users have with the HSE Covid Tracker app, V 1.0.0?

An ancillary analysis also probes the commonalities and differences between Apple and Android users to assess if there are any platform-specific issues that arose and to see how common the profiles are across the two sets of users.

### Structure

In the next section, we discuss the method followed for data gathering and analysis, and then we present our results. Finally, the discussion section focuses on our findings, and potential recommendations for improving the efficiency of the app towards limiting the Covid-19 pandemic.

## Methodology

In order to ensure that the data for the analysis was representative of real-life concerns, the focus was on naturally occurring reviews only. There was initially no discussion/issue forum on the HSE Contact Tracker’s GitHub page [9], but user reviews are available on the Google Play store and the iTunes App store and these reviews been used by other researchers in similar analyses [10] [11] [12]. Hence, a python program was developed to scrape reviews of the HSE’s Covid Tracker app from both the Google Play store and the iTunes App Store. This script was executed on the 13th of August 2020 and scraped all reviews up to that date. It should be noted that at this point in time all the reviews were for version 1.0.0 of the app.

The reviews, thus scraped, were populated into a.csv sheet, which was further converted into.xlsx format for ease of analysis. This spreadsheet is made available for interested readers at [13]. The extracted file was cleaned of duplicates, sorted alphabetically, and contained the following fields:

- User name;
- Time stamp;
- Review id (unique identity);
- The number of stars given to the app in the review (the user’s rating);
- The number of “thumbs-up” from other viewers;
- The associated textual review.

### Analysis

The analysis process in this research involved coding user reviews into 7 aspects, henceforth called pillars: General Characteristics, Usability, Functional Effectiveness, Performance, Data Protection, Autonomy (of users), and Overall-Perception (generic comments). These pillars were derived and refined through a five-phase process, as part of a wider Covid-19 Rapid Response project (“Covigilant”) funded by Science foundation Ireland [5]:

- A bottom-up, inductive approach was taken, where individual contact tracing applications were evaluated for derivation of important app characteristics;
- Cluster analysis [14] was then applied to these characteristics, creating an organizational structure, based on distinct super-categories (or pillars);
- A top-down, deductive approach was carried out in parallel, where the academic/gray literature on (health) app evaluation was used to identify frequently occurring aspects-of-interest across the reviewed papers. For example, van Haasteren et al. [15] identified autonomy as a central concern. Similarly, Vokinger [16] proposed a “user control/self determination” domain in his app assessment framework.
- The results of the top-down and bottom-up analyses were aggregated into a consolidated set of pillars and a pillar advocate identified from the wider Covigilant team for each resultant pillar.
- A devil’s advocate session was undertaken across the team, where pillar advocates had to defend their pillar for relevance and for (sufficient) orthogonality with respect to the other pillars.

Manual sentiment analysis was subsequently performed against the 7-pillar evaluation framework described above. These pillars are referred to in the article by their acronyms: General Characteristics (GC), Overall-perception comment (O), Functional Effectiveness (FE), Usability (U), Data Protection (DP), User Autonomy (A), Transparency (T), and app Performance (P). The analysis was performed manually because, even though there have been huge improvements to automated sentiment analysis in recent years, the precision and recall rates achieved are still not perfect [17] [18] and this would be exacerbated in this instance because here “negative sentiment” aims to capture not just reviews with a negative tone but also quite positive reviews that request a specific refinement or modification.

Each review was randomly allocated to one of four reviewers, the overall allocations to each reviewer being of equal size. These reviewers were tasked with independently segmenting each review into a set of positive and negative user observations, and classifying each of these observations into their appropriate pillar: essentially a form of content analysis that allows “for the objective, systematic and quantitative description of the manifest content of communication” [19] [20]. Here, as mentioned above, “negative user observations” refer to both comments with negative opinions and comments suggesting refinements.

A joint discussion session at the start of the analysis ensured that all reviewers had a common understanding of the seven pillars and the opinions being sought. The subsequent analysis resulted in three new fields being incorporated into the spreadsheet:

- The text segment where a positive, neutral or negative opinion was detected;
- The opinion (positive/negative/neutral) associated with that text segment;
- The associated pillar.

After the coders had individually coded the reviews in this fashion, one author was charged with assessing the entire coding for interpreter drift and inconsistencies in the opinions coded. Interpreter drift is where a coder’s coding drifts over time [21]. For example, in a coder’s initial coding, they may classify a review segment complaining of “the lack of more detailed feedback on the location of cases” as a “usability” issue. But, after fatigue has set in, they may note it as a “performance” issue. In such cases, the author charged with assessing interpreter drift corrected the drift by re-categorizing the latter comments in line with the categorization of the original comments (in the above example, the lack-of-feedback comment would be consistently referred to as “usability”).

In terms of opinion-inconsistencies, there were (14) occasions where a reviewer very obviously ticked the incorrect sentiment. In one case, for example, a user complained of battery drain and the coder incorrectly categorized that opinion as positive. These clear-cut mistakes were also rectified by the author charged with assessing the coding.

**Fig. 2:**
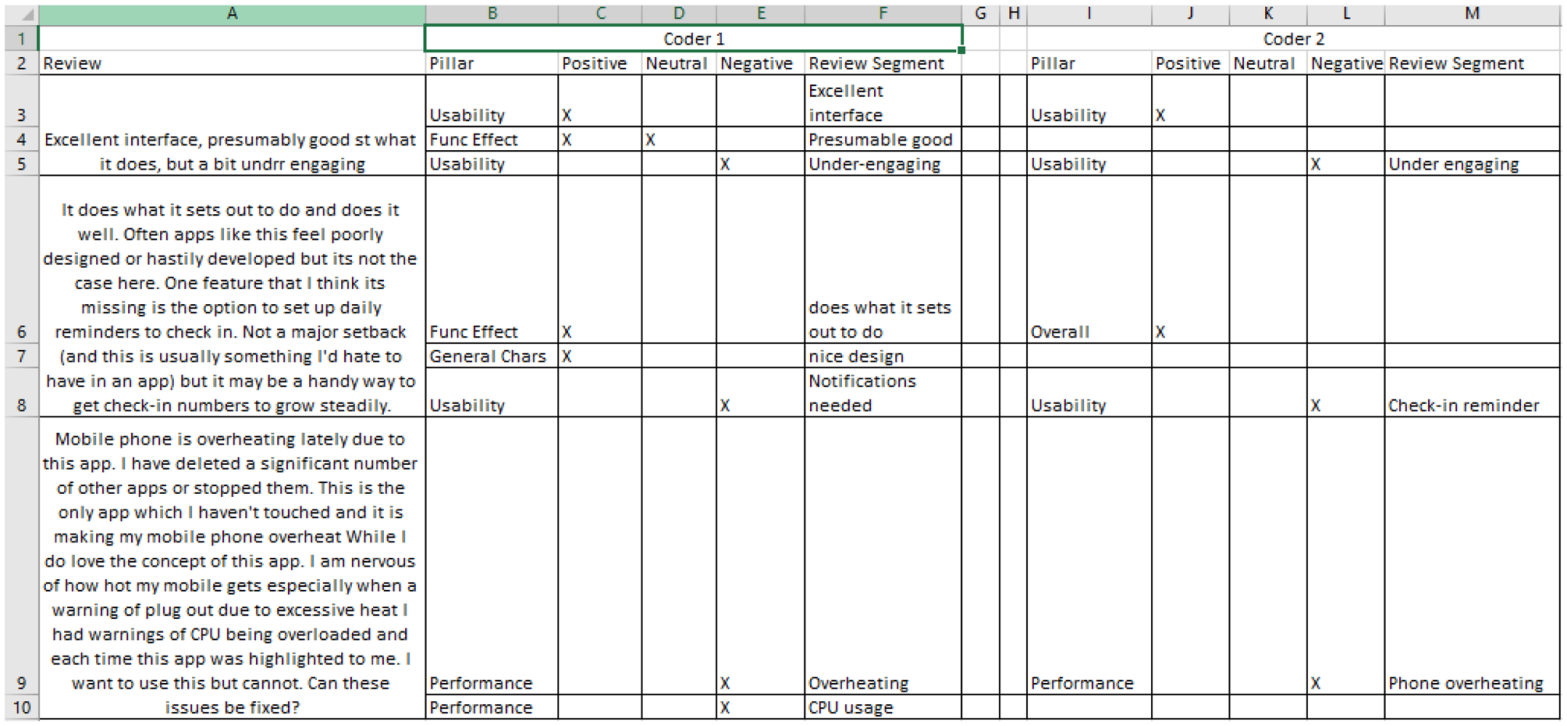
A snippet of the coding spreadsheet

In order to assess inter-coder reliability, approximately one seventh of the reviews were coded by more than one reviewer. Figure 2 presents a snippet of the coding spreadsheet, illustrating several user-comments where more than one reviewer coded the reviews.

Inter-coder reliability could then be assessed using these reviews and the Fleiss’ multi-rater kappa [22, 23]. The protocol was as follows: The text segments both coders identified for each review were re-ordered so that, where possible, they were in the same order across coders. Then the pillars and opinions for each common segment were compared. If they were the same for the same segment, the coders were considered “in agreement.” If either the pillar or sentiment were not the same, the coders were not considered in agreement. Likewise if one of the coders noted a segment that the other did not, then this was considered another disagreement between the coders.

**Fig. 3:**
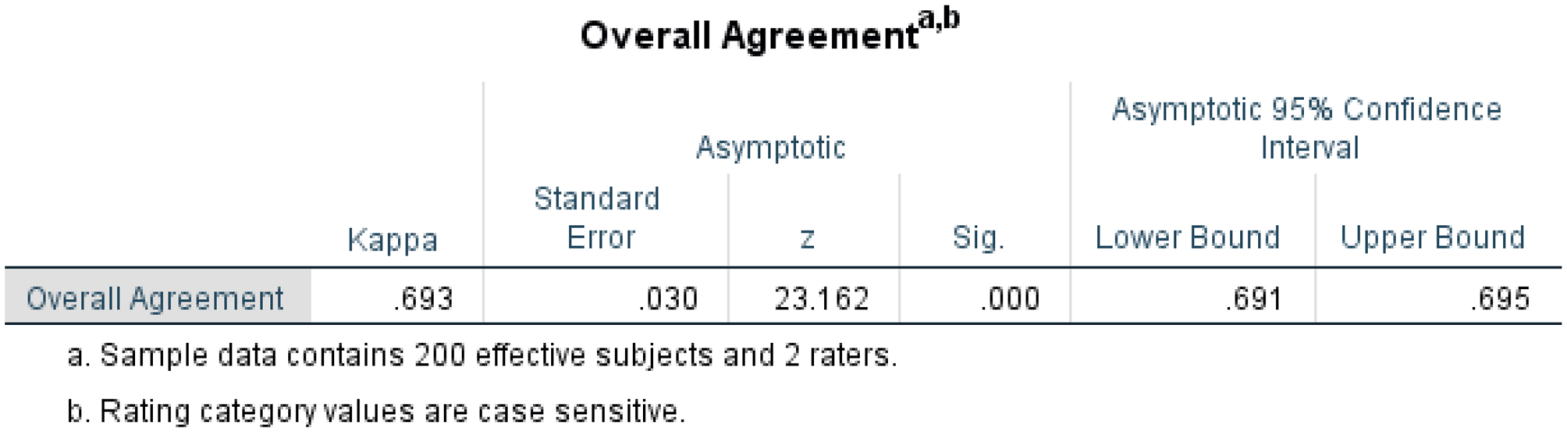
The Fleiss’ kappa results

Figure 3 presents the results of the Fleiss’ kappa analysis performed, revealing a κ of 0.7—a kappa that, according to Landis and Koch [24], suggests a “good” strength of agreement between the coders. The kappa coefficient is statistically significantly different from zero, and the 95% confidence interval for Fleiss’ kappa is .691 to .695.

Subsequently, the individual pillars, opinions, and segments of the entire dataset, as coded by the main coder (not the coders who coded reviews solely for the purposes of the kappa analysis), were analyzed for trends and themes across the reviews: Pillars and opinions were assessed quantitatively to identify the prevalent types of concerns expressed in the data set by the app reviewers. The results of this analysis are now presented.

## Results and findings

In total, 1287 comments were coded, 998 from Android users and 289 from Apple users. Table 1 presents the total number of identified comments per pillar and those totals broken down by positive/negative opinions. In terms of users’ overall perception of the app, the data suggests that they were largely supportive and impressed. Of the 305 comments classified as “overall perception” comments, 274 were positive. This perception was cross platform but slightly higher across Android reviews: Of 223 Android-review comments, 203 (91%) were positive and 71 of 82 (86.6%) Apple-review comments were positive. These “overall” comments focused on the actual implementation itself (“Really well thought out,” “Brilliant App,” “Well implemented,” “it does what it does well”) and, to a lesser degree, on the concept of the app (“its a no brainer,” “great idea”). The ratio of implementation to concept comments was approximately 5:1 in the reviews.

**Table 1:** Results and findings

Apple + Android	Total	Positive	Negative	Negative corrected
Overall perception	305	274	16	16
Usability	390	96	260	206
Performance	448	29	410	0
Data protection	44	32	11	11
Functional effectiveness	70	14	51	51
General characteristics	7	2	2	2
Transparency	23	14	9	9
Sum	1287			

As Table 1 shows, most of the “negative” comments were aimed at performance and usability. The prevalence of performance comments can largely be explained by an Android battery-drain issue that arose on August 8, where one of the Exposure Notification Service libraries provided by Google was updated (by Google) and that update caused significant battery drainage, overheating, and intensive CPU usage, related to the app. Of the 448 performance comments, 444 were from Android users. The hundred sixty-one contained the word “drain,” 53 contained the word “heat,” and five contained the word “CPU.”

So essentially all the negative performance comments were due to this Google update. If this issue were excluded from the analysis, the performance comments would have been entirely positive. However, this seems to have been a very serious issue for Android phone users: Of the 365 reviews associated with these comments 102 mentioned the word “uninstall,” suggesting that well over a quarter of those who complained about the issue considered (at least) uninstalling the app.

Interesting also are the data protection and transparency pillars. Users seem to perceive that the HSE has done well on both fronts with 32 of 44 data-protection comments having a positive perspective and 14 of 23 transparency comments having a positive perspective. This trend was consistent across both Apple and Android devices.

### Usability comments

**Fig. 4:**
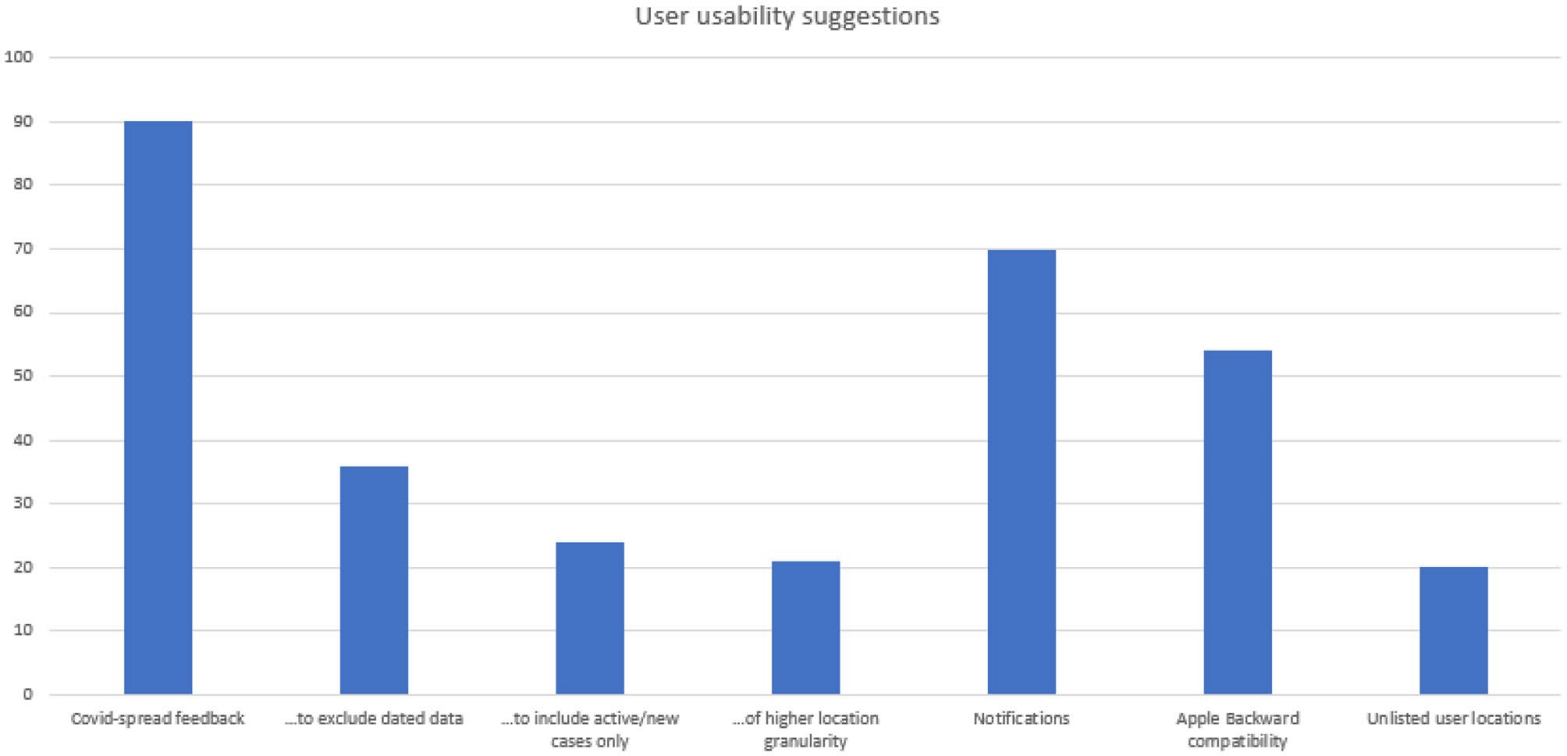
A graph depicting the usability suggestions given by users

The prevalence of usability issues across the reviews is to be expected given the forums involved: Users are most typically interested in usability [25]. Their usability feedback is detailed in Fig. 4.

The main usability issue, as determined by the number of user suggestions (90), was around the feedback provided by the app regarding the occurrence of Covid across the country. Version 1 of the app focused on the number of cases in total at national and county level. Users felt that feedback on Covid cases should focus on a more recent time range (36) on active or newly found cases only (24) and on finer granularity, in terms of geographical location (21). Of this latter category, seven reviewers suggested that hotspots be identified, but this would be difficult in terms of maintaining the current privacy standards the app embodies.

An interesting idea that arose in two reviews was that the app should also report on the number of close contacts users had per day, where the user could get daily feedback and thus try to minimize that “score” over time. This is analogous to the gamification concept of “streaks” [26] where a user might aim to keep their number of close contacts below a certain daily threshold, over time, and thus continue their “streak.”

Another prevalent theme in the usability pillar was a desire for notifications for daily check-ins, where the user self-reports their health status to the app, ideally on a daily basis. Seventy comments requested this enhancement or expressed dissatisfaction at it not currently being available in the app.

A surprising finding was that 20 users complained that a town or area was not available for selection when they were profiling themselves during app setup. Often this was their own town/area, but in five cases reference was made to the exclusion of the six counties in Northern Ireland. Another user noted that it would be interesting to have the possibility of recording two areas, where a user works in one location but lives in another.

Finally, iPhone users complained, in significant numbers, about the app’s inability to work on older versions of iOS or older iPhones (the iPhone 6 particularly). Overall, 54 of the 289 apple comments were targeted as this issue: by far the most prevalent focus of iPhone users’ concerns. This is unfortunate because it represents a significant number of potential users who want to install the app but cannot. In addition, as in the case of the battery-drain issue, this is entirely outside of the HSE’s/NearForm’s control: For this to be addressed, Apple would need to incorporate backward compatibility into the associated operating systems. The last column of Table 1 shows the “negative” sentiment corrected for these outside-of-the-HSE’s-NearForm-control issues.

## Discussions

In general, the public seems positively predisposed to the HSE’s Contact Tracking app. The overall-perception comments are largely supportive and, on the critical aspects of data protection, and transparency, public opinion seems favorable, as assessed by the Google Play Store and iTunes App reviews.

Below that positivity though, there are some prevalent user requests or concerns that should be addressed. The most prevalent platform-specific concerns (and indeed two of the three most prevalent concerns across platforms) are outside of the developer’s control. The battery drain issue was caused by an update to an underpinning Google library, and was remedied quickly, although not before substantial reputational damage had been done in terms of the public’s perception of the app. Likewise, the backward-compatibility issue for older versions of iOS is a significant issue for potential users and can only be addressed by Apple.

Something that the HSE/NearForm have already worked on, in their newer versions of the app, is the information that the app gives about the spread of the disease. They have tackled the desire for more timely information with information now provided on new cases within the last day, 2 weeks, and 2 months at a national level. They have also expanded the information available at county level, where again users can see the new cases for the last day, 2 weeks, and 2 months.

Additionally, they have increased the granularity of information presented by the app in terms of geographical location: that information is now presented in line with one reviewer’s suggestion that the facility be offered at electoral division level, a facility previously offered by the Irish Central Statistic’s office (https://census.cso.ie/covid19/: however, the statistics presented on this website are for before the 20th of June 2020 only, and so were out of date).

Another aspect prevalent in the reviews, and one that might facilitate increased user engagement, was the use of daily notification alerts to remind users to check-in with their health status. This, combined with the improved disease-spread feedback provided by the app, and maybe gamification of aspects like recording daily close contacts, may encourage users to retain the app and engage with it for longer time periods [26].

More holistically, the *path of this research* argues for greater analysis of user feedback because of the apps’ requirement for high user take-up and high user retention. Ideally, this analysis should lead to remedial action where possible—a position implicitly adopted by the HSE in more recent times by providing a developer-moderated GitHub page, where users can request changes and developers can respond (https://github.com/HSEIreland/covid-tracker-app/pulls).

*The actual findings of this research* suggest that the contact tracing apps, which are largely passive in nature should have proactive notifications, and that they should provide timely and detailed disease-spread information to the users, to keep those users more engaged. Finally, the health authorities should work closely with the GAEN service providers to lessen backward-compatibility issues and to ensure that any critical problems that arise in that GAEN service are addressed as quickly as possible, before they lead to user disenfranchisement.

### Limitations and threats to validity

In terms of reliability, manual analysis was done by different coders, so there may be a bias or an inconsistency in that coding. To mitigate against these possibilities, the four coders were all given an introductory session where 15 illustrative examples were coded, and discussion undertaken to form a common understanding. The inter-coder reliability assessment undertaken in this study suggests that this introductory session largely achieved its goal, as discussed in “Methodology,” and that the reliability of the analysis was good.

A construct validity issue [27] is that the data obtained may not be totally correct: User opinions may be informed by hearsay and users are not always in an ideal position to report on quality aspects like data protection, or performance [28]. To mitigate against this, the analysis focused on opinions that were more pervasive across the dataset and brought to bear considerable knowledge of the app itself, the researchers having studied it as part of the wider Covigilant project goal, to derive the pillars beforehand [5].

Another external validity issue [27] is that our dataset is only a very small sample of the user population. Even considering that a large number of users may have uninstalled the app after the Android battery issue, and the currently quoted download figure of over two million is thus somewhat inflated, it is likely that there are over 1.3 million active users (this is quoted in the app’s User Interface). While a sample of 1287 comments is small by comparison, it can be considered representative [29]. But a more serious, related, external validity issue is that the sampling protocol in this case is self-selecting, where only vocal reviewers (those that left reviews) are included in the data-set. Scientists have long established that self-selection participation can be problematic across a range of subject matter [30, 31], and it is likely that this is no exception. Particularly, it seems likely that the silent majority are quite happy with the app and that the issues noted are not as emphasized across the user-base, as the data would suggest.

In addition, the population from which these self-selecting reviewers are drawn is not entirely representative of the population. A report commissioned by the Department of Health in Ireland showed lower smartphone ownership in terms of “older age groups… specific occupations (those engaged in home duties), materially deprived groups and in border areas” [32]. Given that COVID-19 vulnerable groups are skewed in terms of older age adults, the lower take-up of smartphone apps by older adults is a key limitation of the contact tracing mitigation strategy in general. Studies have been conducted that identify this lack of smartphone app adoption by older adults [33].

## Conclusion

This report has focused on sentiment analysis of all the reviews that were available on Google Play and Apple collectively before August 14, 2020, towards helping evolve a better contact tracing application to fight the Corona virus. The results suggest that the app is well perceived and seen as sensitive to data-protection concerns. But the reviews also suggest that notifications should be included, to remind users to lodge their health status every day. Additionally users seemed upset when their location was not available in the app. Finally, efforts should be directed at prompting Apple to make their Exposure Notification Service available to older iPhones and older versions of iOS.

But some of the suggestions made by users have already been addressed in the next version of the Covid Tracker that was launched by the HSE. Specifically feedback on the status of Covid-19 has been made more current in terms of time-span reported and current cases. Additionally, the app now reports on location of cases with greater granularity. This consilience between our results and HSE updates not only strengthens the efficacy of our other findings but also suggests that the health authorities in Ireland are evolving the app in a direction cognizant and sympathetic to user concerns.

While the analysis proved to be helpful in understanding the public’s opinions regarding the HSE’s Covid Tracker, an automated analysis of users’ concerns using artificial intelligence could also be developed [34]. This would facilitate understanding wider public opinion about the app over larger datasets, in a much more time-sensitive manner.

**Fig. 5:**
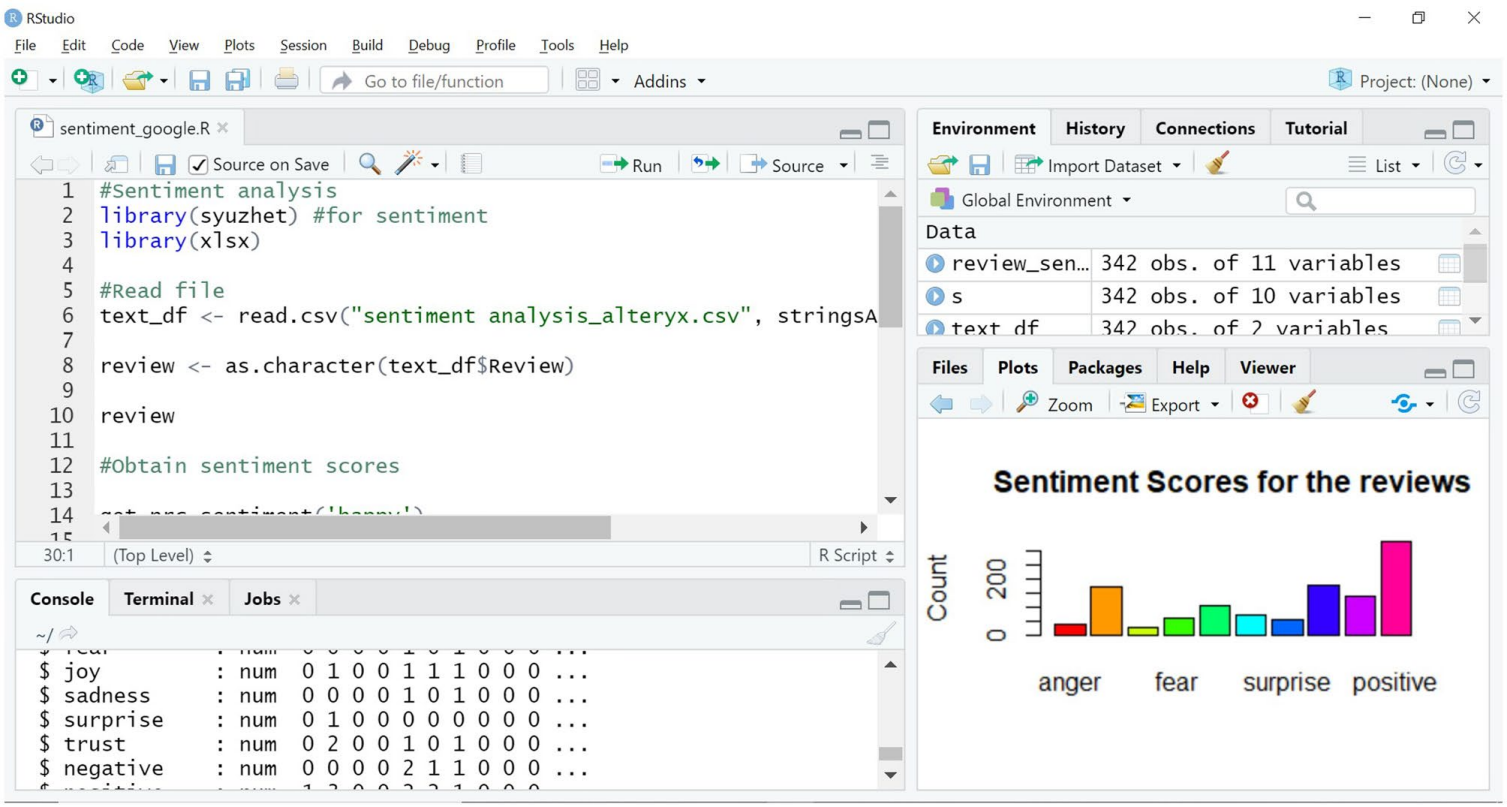
Sentiment analysis using R Studio

Our future work will be in this direction, and already we have trialed an initial approach. The existing reviews, scraped and extracted into a.csv file, have been cleaned for special characters and unnecessary symbols using Alteryx. Sentiment analysis has been done using R Studio, as shown in Fig. 5.

In this context, sentiment analysis is the computational task of automatically determining what feelings a writer is expressing in text [35]. Sentiment is often framed as a binary distinction (positive vs. negative), but it can also be more fine-grained, like identifying the specific emotion an author is expressing (like fear, joy, or anger) [18].

However, we are conscious that sentiment analysis alone may only help us focus our analysis on the opinions of users [35, 36]. It will not identify the area of interest, identify the users’ specific issues or, indeed, determine the prevalence of those issues across the datasets. So additional analysis may be required to facilitate identification of the users’ issues. Cluster analysis for example [14] could be used to group together dissatisfied reviews into relevant and prevalent themes/topics of focus.

## Data Availability

https://www.lero.ie/sites/default/files/journal%20copy%20of%20scraped%20and%20cleaned%20data.xlsx

## Code availability

The Python scripts employed to scrape the web pages can be obtained from the authors, on request.
